# Utilizing POCUS for the Identification and Management of PICC Line-Associated Cardiac Tamponade

**DOI:** 10.24908/pocusj.v10i02.18439

**Published:** 2025-11-17

**Authors:** Pedro Jose Cruz Guzman, Karen Lidsky, William Hanna

**Affiliations:** Department of Pediatric Critical Care, Cleveland Clinic Children's, Cleveland, Ohio, USA

**Keywords:** Pediatric critical care, PICC line, POCUS, Pericardial effusion, Point of care ultrasound, Cardiac tamponade

## Abstract

Peripherally inserted central catheters (PICCs) are commonly used in pediatric patients, but rare complications such as cardiac tamponade can occur. This case report describes a 5-month-old ex-premature infant who developed cardiac tamponade associated with PICC line malposition. Point of care ultrasound (POCUS) was used to diagnose and effectively manage the condition peri-arrest via emergent pericardiocentesis. The report highlights the role of POCUS in the prompt identification and treatment of hemodynamic deteriorations and posits potential utilization of POCUS as a tool in preventative surveillance.

## Introduction

Peripherally inserted central catheters (PICCs) are extensively utilized in pediatrics for long-term therapies including nutrition, chemotherapy, and antibiotics due to their ease of insertion, low insertion related complications, and lower costs [1,2]. Despite their advantages and safety, PICCs still remain prone to occlusion, thrombosis, infections, migrations, and—rarely— pleural or pericardial effusions [3]. Cardiac tamponade related to PICC line malposition is a rare yet serious complication, with neonatal studies suggesting an incidence ranging from 0.049% to 1.5% and a mortality as high as 50%. Despite its rarity, the consequences can be devastating and require prompt diagnosis and management [3–5].

Within acute care pediatric settings, the use of point of care ultrasound (POCUS) continues to expand beyond standard procedural applications such as central venous access. More recent applications in the literature are extensive, including effective placement and surveillance of PICC line tip position and as a diagnostic tool in better characterizing and managing undifferentiated shock states [6–10].

This case report describes a pediatric case of cardiac tamponade related to PICC line malposition and highlights the unique role of POCUS for both acute diagnosis and management, and potentially as a mode of surveillance in early diagnosis of these complications.

## Case presentation

A 5-month-old ex-34-week, infant girl was initially admitted to an outside hospital with new onset acute liver failure. Her initial clinical course following transfer involved supportive care including mechanical ventilation and nutritional support along with an extensive diagnostic evaluation, being managed in the pediatric intensive care unit by a multidisciplinary team including pediatric gastroenterology, hematology, nephrology, and palliative care.

On day 3 of admission, a decision was made to place a PICC line given the need for ongoing antibiotics and total parenteral nutrition. Access was obtained via the right basilic vein using fluoroscopic guidance, placing a 4 French double-lumen catheter that was cut at the 15 cm mark. The tip of the PICC was confirmed with fluoroscopy and post-PICC chest x-ray to be in an appropriate position (SVC/RA junction), with the patient transferred back to the pediatric intensive care unit in stable condition.

Her respiratory status improved within the next 4 days following PICC placement, leading to her extubation. Within the subsequent 24 hours, she developed tachycardia, tachypnea, hypoxemia and decreased perfusion with no improvement, despite using non-invasive ventilation (HFNC, BiPAP). This necessitated re-intubation that resulted in a very brief period of improved perfusion and vital signs. Within the next 10 minutes, however, she became acutely hypotensive of unclear etiology, degenerating into pulseless electrical activity (PEA) arrest requiring cardiopulmonary resuscitation (CPR). During a scheduled compression pause within the first 10 minutes, POCUS was performed by an experienced pediatric intensivist utilizing the GE Venue Ultrasound system (Chicago, Illinois, USA) with 6S phased array transducer over the subxiphoid area. This generated a cardiac subcostal window that exhibited a massive circumferential pericardial effusion with right ventricle collapse throughout the cardiac cycle and pseudo-PEA. A 21 g 7 cm needle was used with syringe and direct image guidance to evacuate 50 mL of cloudy fluid from the pericardial space. This caused the immediate return of spontaneous circulation with minimal residual pericardial effusion. A temporary 20 g 1 ¾ inch catheter was then placed with a subsequent brief period of clinical stabilization. Shortly after establishing additional arterial and venous access via intraosseous line, she was noted again to show signs of severely low output state. POCUS imaging confirmed the re-accumulation of a large circumferential pericardial effusion that was evacuated in similar fashion with stabilization of hemodynamics. With concern for PICC line malfunction being actively raised, a bedside cardiac bubble study was then performed through the PICC line. This revealed bubbles extravasating from the right atrium directly into the pericardial space (see Supplementary Material - S1). Visualization of the PICC line with the catheter in the low right atrium and projecting in the direction of the tricuspid valve (see Supplementary Material - S2) suggested the malpositioned PICC line as the cause of a likely intracardiac perforation. She developed a second PEA arrest lasting one minute, which occurred immediately after placement of the definitive pericardial drain. Surveillance POCUS confirmed that the event was unrelated to pericardial fluid reaccumulation. The PICC line was removed and post-cardiac arrest protocols were followed.

## Discussion

Our case represents one of the rarest and most severe complications associated with PICC line placement and highlights the unique role that POCUS may play in diagnosing, managing, and potentially preventing PICC line-associated cardiac tamponade.

Recent pediatric and neonatal evidence-based guidelines Recentcognize the value and role of POCUS in further characterizing low cardiac output states, including assessment of ventricular function, intravascular volume status, and life-threatening conditions such as pulmonary hypertensive crises and pericardial tamponade [9]. Within pediatrics, focused cardiac ultrasound has been shown to influence the practitioner's characterization of hemodynamics, although robust clinical outcome data remains lacking. However, recent adult literature suggests that appropriate utilization of POCUS in shock may positively impact mortality, lactate clearance, and the duration of vasoactive needs, among other parameters [11]. This case highlights a clear proof-of-concept instance of POCUS use in pediatrics as a time sensitive, question-focused adjunct that directly affected patient outcome. POCUS utilization helped with characterizing and treating tamponade, isolating the specific etiology of the tamponade, and reassessing for recurrence during repeated episodes of clinical deterioration. This instance and others within the literature may serve to further strengthen the claim that POCUS, when used by the right person, for the right indication, and at the right time, can function as a powerful and necessary tool in critically ill pediatric patients [12,13].

Besides recognizing and managing acute clinical changes, POCUS may have an equally important role in surveillance and prevention of PICC-related complications. Prospective studies, such as those by Lin and Katheria, suggest pediatric POCUS use during PICC line placement optimizes PICC tip position and reduces both time for successful placement and need for repeated radiography when compared to standard approaches to placement [14]. Despite optimal placement, it is thought that up to 24% of PICC lines migrate within the first 24 hours following insertion in neonates and small infants. Moreover, while full consensus may not exist regarding optimal catheter tip position, vascular perforation has still been described for catheters with tips outside of the cardiac silhouette. Potential proposed mechanisms include chronic friction, which can result in vascular wall compromise, especially in neonates and small infants whose thin vascular walls and fast heart rates may further predispose them to such events [2,15]. Studies like Trinh HT et al. advocate for the use of emergent bedside POCUS for unexplained clinical deterioration post-PICC placement and to surveil tip position or other subclinical findings twice weekly. While standard recommendations regarding POCUS surveillance are lacking, given the high mortality of such complications and minimal risk and time associated with POCUS surveillance, we would agree with regular proposed surveillance checks as a preventative measure in high-risk neonates and infants [5].

## Conclusion

This case serves as a reminder of the potential hazards associated with PICC lines in pediatric patients and the potential that POCUS offers as a lifesaving diagnostic and therapeutic tool for PICC line-associated cardiac tamponade. The adoption of POCUS both during clinical deteriorations and potentially in routine clinical surveillance for higher risk patients may improve patient safety and healthcare outcomes.

## References

[1] Moureau N, Chopra V. Indications management and complications of PICC lines: essential information for clinical practice. J Vasc Access 2016;17(5):453–464 27516141

[2] Paterson RS, Chopra V, Brown E, Kleidon TM, Cooke M, Rickard CM, Bernstein SJ, Ullman AJ. Selection and insertion of vascular access devices in pediatrics: a systematic review. Pediatrics 2020;145(Suppl 3):S243–S268. doi: 10.1542/peds.2019-3474H.1 32482738

[3] Zareef R, Anka M, Hatab T, El Rassi I, Yunis K, Bitar F, Arabi M. Tamponade and massive pleural effusions secondary to peripherally inserted central catheter in neonates—a complication to be aware of. Front Cardiovasc Med 2023;10:1092814. doi: 10.3389/fcvm.2023.1092814 36873398 PMC9981636

[4] Wang J, Wang Q, Liu Y, Lin Z, Janjua MU, Peng J, Du J. The incidence and mortality rate of catheter-related neonatal pericardial effusion: a meta-analysis. Medicine (Baltimore) 2022;101(47):e32050. doi: 10.1097/MD.0000000000032050 36451499 PMC9704876

[5] Trinh HT, Nguyen TT, Nguyen TT. Cardiac tamponade due to pericardial effusion following peripherally inserted central catheter: a single-institution case series. Cureus 2024;16(3):e56403. doi: 10.7759/cureus.56403 38638757 PMC11025877

[6] Cui Y, Liu K, Luan L, Liang P. Delayed cardiac tamponade diagnosed by point-of-care ultrasound in a neonate after peripherally inserted central catheter placement: a case report. World J Clin Cases 2021;9(3):602–606. doi: 10.12998/wjcc.v9.i3.602 33553397 PMC7829730

[7] Lin SY, Chiang MC, Wu WH, Wu IH, Lai MY, Chu SM, Lien R, Hsu KH. Point-of-care ultrasound (POCUS) for tip localization of neonatal peripherally inserted central catheter (PICC): a prospective study. Pediatr Neonatol 2023;S1875-9572(23):00220–6. doi: 10.1016/j.pedneo.2023.07.008 38114415

[8] Singh Y, Tissot C, Fraga MV, et al. International evidence-based guidelines on Point of Care Ultrasound (POCUS) for critically ill neonates and children issued by the POCUS Working Group of the European Society of Paediatric and Neonatal Intensive Care (ESPNIC). Crit Care 2020;24:65. 32093763 10.1186/s13054-020-2787-9 PMC7041196

[9] Conlon TW, Nishisaki A, Singh Y, Bhombal S, De Luca D, Kessler DO, Su ER, Chen AE, Fraga MV. Moving Beyond the Stethoscope: Diagnostic Point-of-Care Ultrasound in Pediatric Practice. Pediatrics 2019;144(4):e20191402. doi: 10.1542/peds.2019-1402 31481415

[10] Arnoldi S, Glau CL, Walker SB, Himebauch AS, Parikh DS, Udeh SC, Weiss SL, Fitzgerald JC, Nishisaki A, Conlon TW. Integrating Focused Cardiac Ultrasound Into Pediatric Septic Shock Assessment. Pediatr Crit Care Med 2021;1;22(3):262–274. 33657611 10.1097/PCC.0000000000002658

[11] Miller A, Kaplansky T, Baldwin C, Fifer K, Joshi N. The impact of point-of-care ultrasound-guided resuscitation on clinical outcomes in patients with shock: a systematic review and meta-analysis. J Intensive Care Med 2023;38(5):555–567. doi: 10.1177/08850666221099242 39298556

[12] Doniger SJ, Ng N. Cardiac point-of-care ultrasound reveals unexpected, life-threatening findings in two children. Ultrasound J 2020;12:4. doi: 10.1186/s13089-020-0154-3 32016667 PMC6997318

[13] Raimondi F, Rodriguez Fanjul J, Aversa S, Chirico G, Yousef N, De Luca D, Corsini I, Dani C, Grappone L, Orfeo L, Migliaro F, Vallone G, Capasso L; Lung Ultrasound in the Crashing Infant (LUCI) Protocol Study Group. Lung ultrasound for diagnosing pneumothorax in the critically ill neonate. J Pediatr 2016;175:74–78.e1. doi: 10.1016/j.jpeds.2016.04.018 27189678

[14] Katheria AC, Fleming SE, Kim JH. A randomized controlled trial of ultrasound-guided peripherally inserted central catheters compared with standard radiograph in neonates. J Perinatol 2013;33(10):791–794. doi: 10.1038/jp.2013.58 23765173

[15] Gupta R, Drendel AL, Hoffmann RG, Quijano CV, Uhing MR. Migration of central venous catheters in neonates: a radiographic assessment. Am J Perinatol 2016;33(6):600–604. doi: 10.1055/s-0035-157034 26731179

